# A newborn with normal IgM and elevated IgG antibodies born to an asymptomatic infection mother with COVID-19

**DOI:** 10.18632/aging.103346

**Published:** 2020-09-11

**Authors:** Wenqi Gao, Zhifang Deng, Lingkong Zeng, Yuan Yang, HongJian Gong, Jue Liu, Han Xiao

**Affiliations:** 1 Institute of Maternal and Child Health, Wuhan Children’s Hospital, Tongji Medical College, Huazhong University of Science and Technology, Wuhan, China; 2 Department of Pharmacy, The Central Hospital of Wuhan, Tongji Medical College, Huazhong University of Science and Technology, Wuhan, China; 3 Department of Neonatology, Wuhan Children’s Hospital, Tongji Medical College, Huazhong University of Science and Technology, Wuhan, China

**Keywords:** COVID-19, COVID-19 antibody, neonate

## Abstract

Pregnant women are susceptible population of COVID-19 which are more likely to have complications and even progress to severe illness. Pregnancy with COVID-19 and neonates are rarely reported. We report a newborn with normal IgM and elevated IgG antibodies born to an asymptomatic infection mother with COVID-19. We assessed whether there was intrauterine vertical transmission potential of COVID-19.

## Dear Editor

We reported a newborn with normal IgM and elevated IgG antibodies born to an asymptomatic infection mother with coronavirus disease 2019 (COVID-19). In our present case, the mother of the neonate was a 30-year-old pregnant woman. There were no confirmed or suspected cases of COVID-19 in her family. She denied having a history of exposure to COVID-19 patients. She was pregnant for the first time. She claimed that she had never had syphilis, hepatitis b, AIDS and other infectious diseases.

March 4, 2020, the gestation pregnant woman was 39 weeks pregnant. Color ultrasound indicated that the fetus was in the breech position with the umbilical cord around the neck. At 02:05 on March 6, 2020, the pregnant woman went to Wuhan Central Hospital for treatment due to excessive amniotic fluid and umbilical cord around the neck. There were no typical symptoms of COVID-19, such as fever and cough, in this pregnant woman. Thoracic computerized tomography scan revealed no abnormality. The nucleic acid test of pharyngeal swab showed positive, and the results of serum IgM and IgG antibody (colloidal gold method) were weak positive and strong positive, respectively, suggesting that the pregnant woman might be an asymptomatic infection case of COVID-19. Blood tests showed lymphocytes (0.82×10^9^/L, normal: 1.1-3.2×10^9^/L) reduced. She was hospitalized for suspected viral pneumonia.

On admission, her body temperature was 36.4°C and her blood pressure was 108/65 mmHg, with respiratory rate of 20 breaths per minute, pulse of 76 beats per minute. Cardiopulmonary function was normal, and there was no edema in the lower limbs. No intrauterine distress was presented throughout the pregnancy. Amniotic fluid slant overloaded, without amniotic fluid pollution. Fetal heart monitoring showed no abnormalities, and the fetal heart rate was 140 bpm. Emergency cesarean section was operated for pregnant women. The pregnant woman wore an N95 mask throughout the operation, without cough or produce sputum.

At 14:00 on March 6, 2020 a baby girl was born, weighted 3,460g. Apgar scores at 1 and 5 minutes were 9 and 10, respectively. The baby did not get groan, fever, cough, and vomit. The baby had a ruddy face and a powerful cry. Since there was no isolation ward in the neonatal department of Wuhan Central Hospital, the neonate was transferred to COVID-19 children's designated hospital-Wuhan Children's Hospital immediately after 3 hours of birth. The newborn could eat normal breast milk. The newborn's mental response was good, with blood oxygen saturation maintaining more than 92%. Her body temperature and body length were 36.9°C and 50 cm, with respiratory rate of 36 breaths per minute, pulse of 135 beats per minute.

**Figure 1:**
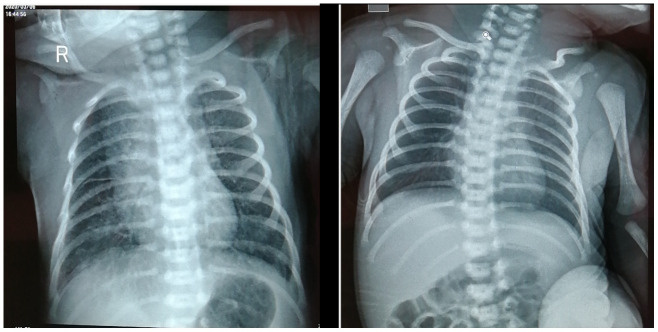
Chest X-ray of neonate on March 6^th^, 2020 (**A**) and March 12^th^, 2020 (**B**).

Laboratory reports of this infant were negative, including toxoplasma, herpes simplex virus 1/2, cytomegalovirus (CMV), and rubella virus. Neutrophils percentage (69.5%, normal: 31% ~ 52%), basophilic cells percentage (0.70%, normal: 0% ~ 0.6%), neutrophils total (13.45%, normal: 3.9% ~ 9.4%) were all increased. Liver dysfunction (AST 92U/L, normal: ≤41 U/L), creatine kinase (189U/L, normal: 30 ~ 170 U/L), creatine kinase isoenzyme MB (60U/L, normal: 0 ~ 24U/L) levels increased. Procalcitonin increased (0.880ng/ml, normal: ≤0.05ng/ml). IL-6 (49.00pg/ml, normal: 0 ~ 20.9pg/ml) and IL-10 (6.28pg/ml, normal: 0 ~ 5.9pg/ml) increased. Renal function and electrolytes were normal. Figure 1A Chest X-ray showed enhanced lung veins, reticular and patchy shadows, and no abnormalities in heart and palate (image). She was closely monitored in isolation, treated with a nourishing cardiac muscle and a spray of interferon. Intravenous injection of penicillin G (15wu q.d, intravenous bolus) and vitamin K1 (1mg q.d, intravenously) were used as antibiotics and to prevent coagulation disorders.

March 7, 2020 the second day after the surgery, the newborn was in good condition and the mother's vital signs were stable. Baby was closely monitored and given 30ml of formula every three hours.

From March 7 to March 12, 2020 the newborn's vital signs were stable, the blood oxygen saturation maintaining above 90%, and there was no apnea or vomit. On March 9, the nucleic acid test of neonatal COVID-19 pharyngeal swab was negative, however, and serum COVID-19 IgM and IgG antibodies were normal and strong positive, respectively. The blood routine, liver function, calcitonin, and creatine kinase levels all returned to normal. Chest X-ray of neonate showed a few flaky shadow, with no abnormality in heart and palate. Compared with the chest X-ray on March 6, the Chest X-ray Figure 1B of March 12 revealed that most of the lung lesions were absorbed. She did not receive any special treatment since March 12. On March 17, laboratory tests and chest radiographs were normal, the nucleic acid test of neonatal COVID-19 pharyngeal swab and serum IgM were both negative. The newborn was discharged from hospital on March 18, 2020.

June 17, 2020 about one hundred days after the neonate was born, we detected the serum COVID-19 IgM and IgG antibodies of mother and infant again. The serum COVID-19 IgM and IgG antibodies of mother were still negative and positive, while the IgG antibody of infant decreased rapidly and changed into negative.

Since December 2019, pneumonia caused by SARS-CoV-2 has become a highly contagious disease. As of April 27, 2020, a total of 2,878,196 COVID–19 cases and 198,668 related deaths have been confirmed [1]. Since COVID-19 was a brand new infectious disease and the immunological detection reagent has just been developed, there were few reports of COVID-19 vertical transmission tracing in pregnant women. Here, we reported a newborn with normal IgM and elevated IgG antibodies born to an asymptomatic infection mother with COVID-19. The viral nucleic acid of the pharyngeal swab in newborn was negative. The pathogenic test found that the serum of IgM of the newborn was normal, and IgG was strongly positive. In general, after the body infecting with pathogenic microorganisms, the immune system carries out immune defense against the virus and produces specific antibodies. Specific IgM may indicate a current or recent infection. IgG is the main antibody produced in the immune response, indicating that the disease has entered the recovery period or the presence of previous infection [2–3].

When the infant was born, the level of IgG antibody in her serum was similar to that of her mother, both strong positive. However, the infant IgG antibody decreased rapidly and turned negative after about one hundred days, while the maternal IgG antibody remains at a high level. According to the examination results of mother and neonate, we suspected that, on the one hand, the neonate might acquire the IgG antibody from mother via placenta. The infant did not produce IgG antibody, therefore, the level of IgG antibody decreased remarkably at time passed. On the other hand, the mother might expose to small numbers of virus before, and was an asymptomatic infection case of COVID-19, thus, the neonate might be in the recovery period of COVID-19 at present. Because of the children's immune systems were underdeveloped, the level of IgG antibody in infant reduced rapidly.

There were several limitations in this study. 1) Lack of nucleic acid detection results in breast milk and placenta; 2) Our present report include the single case, more information was need to confirm the observation.
